# Coordinating a Supply Chain with a Loss-Averse Retailer and Effort Dependent Demand

**DOI:** 10.1155/2014/231041

**Published:** 2014-07-23

**Authors:** Liying Li, Yong Wang

**Affiliations:** ^1^Science College, Chongqing Jiaotong University, Chongqing 400074, China; ^2^School of Economics and Business Administration, Chongqing Key Laboratory of Logistics, Chongqing University, Chongqing 400044, China

## Abstract

This study investigates the channel coordination issue of a supply chain with a risk-neutral manufacturer and a loss-averse retailer facing stochastic demand that is sensitive to sales effort. Under the loss-averse newsvendor setting, a distribution-free gain/loss-sharing-and-buyback (GLB) contract has been shown to be able to coordinate the supply chain. However, we find that a GLB contract remains ineffective in managing the supply chain when retailer sales efforts influence the demand. To effectively coordinate the channel, we propose to combine a GLB contract with sales rebate and penalty (SRP) contract. In addition, we discover a special class of gain/loss contracts that can coordinate the supply chain and arbitrarily allocate the expected supply chain profit between the manufacturer and the retailer. We then analyze the effect of loss aversion on the retailer's decision-making behavior and supply chain performance. Finally, we perform a numerical study to illustrate the findings and gain additional insights.

## 1. Introduction

Coordination among members of a supply chain is an essential strategic issue [1]. With supply chain coordination, the upstream member offers a set of appropriate contract parameters to the downstream member such that the self-profit maximizing objective of the latter is aligned with the objective of the whole supply chain during decision making. Major coordination mechanisms include buyback contracts [2], quantity-flexibility contracts [3], quantity-discount contracts [4], sales-rebate contracts [5], and revenue-sharing contracts [6]. Cachon [7] provides good reviews of this literature.

Promotional activities have become increasingly more common in the service industry. FedEx and UPS provide transportation cost discounts to attract customers; A&F Clothiers and American Eagle make shelf space for specific clothing items available for longer periods; Wal-Mart and Target often try to stimulate demand for specific types of foods by offering free trials [8]. Generally, a retailer can increase demand of the goods by applying promotional effort such as advertising, sales team, free gifts, discount on sales price, and attractive shelf space [1]. All these efforts are costly. If the manufacturer (supplier) does not provide sufficient incentives, then the retailer will have no motivation to enhance effort level.

Most studies on supply chain management assume that managers are risk neutral; that is, they make decisions to maximize expected profits. However, many empirical studies and observations on managerial decision making under uncertainties show that the decision-making behavior of managers deviates from maximizing expected profits and is consistent with loss aversion [9–13]. Loss aversion, which is one of the key features of prospect theory [14], indicates that people are more sensitive to losses than to same-sized gains and that the perception of gains or losses is related to a reference point [15, 16]. Loss aversion is both intuitively appealing and well supported in finance, economics, marketing, and organizational behavior [17, 18].

Incorporating the aforementioned factors, this study investigates the channel coordination issue of a single-period, two-echelon supply chain with a manufacturer, and a retailer facing stochastic demand that is influenced by sales effort. We assume that the manufacturer, as a large diversified firm, is risk neutral, whereas the retailer, as a smaller and less diversified firm, is loss averse. Our major research questions are as follows.

1. Is there a contract that can coordinate a supply chain with a loss-averse retailer and effort dependent demand?
2. Can such a coordinating contract arbitrarily allocate the total expected supply chain profit?
3. What effect does loss aversion have on the optimal order quantity and effort level of the retailer?

The main contributions of this study are as follows. First, we develop supply chain models that incorporate both a loss-averse retailer and sales-effort dependent demand. Second, we show that a distribution-free gain/loss-sharing-and-buyback (GLB) contract alone cannot coordinate the supply chain, whereas a GLB contract combined with sales rebate and penalty (SRP) can achieve coordination. In addition, we discover a special class of gain/loss (GL) contracts that can coordinate the supply chain. Furthermore, we show that the two aforementioned coordinating contracts can arbitrarily allocate the expected supply chain profit between the manufacturer and the retailer. Finally, we discuss the effect of loss aversion on the retailer's decision making and supply chain performance.

This paper is organized as follows. We review related literature in Section 2. Then, the model is formulated and assumptions are proposed in Section 3. We present the contracts that coordinate the supply chain in Section 4. In Section 5, we investigate the coordination efficiency of the contracts.  Section 6 illustrates a numerical example. Finally, conclusions and future research prospects are provided in Section 7.

## 2. Literature Review

In this section, we review only studies that are most relevant to our research. First, we discuss the stream of research that considers supply chain management with sales-effort dependent demand. Cachon [7] demonstrates that both returns policy and revenue-sharing contracts fail to coordinate a supply chain under this setting, and the optimal effort of the retailer is lower than that of the integrated supply chain. Taylor [5] shows that a properly designed returns policy with channel rebates can coordinate the supply chain and achieve a win-win outcome when retailer sales effort influences demand. Cachon and Lariviere [6] show that revenue-sharing contracts cannot coordinate the supply chain, and they present a quantity-discount contract that is related to revenue sharing to achieve coordination. Krishnan et al. [19] show that a buyback contract alone cannot coordinate the supply chain and combining a buyback contract with cost-sharing agreements is the best way to achieve channel coordination. He et al. [20] investigate the channel coordination issue of a supply chain facing stochastic demand that is sensitive to both sales effort and retail price. They find that none of the traditional contracts, such as returns policy or revenue-sharing contracts, can coordinate the supply chain, and only a properly designed returns policy with SRP contract can achieve channel coordination. Tsao and Sheen [8] consider promotion cost as a mechanism to coordinate the supply chain under the promotional efforts of the retailer and sales learning curve. Xing and Liu [21] consider the channel coordination issue of a supply chain wherein an online retailer offers a low price and free-rides a brick-and-mortar retailer's sales effort. Ma et al. [22] also deem marketing effort cost sharing as a critical mechanism to achieve channel coordination. The main difference between our study and the aforementioned literature is that we incorporate loss aversion into supply chain management with sales-effort dependent demand.

Another stream of research closely related to our study deals with loss aversion. Schweitzer and Cachon [12] are believed to be the first to investigate loss-averse newsvendor issues. They find that a loss-averse newsvendor will order less than a risk-neutral newsvendor when shortage cost is negligible. Wang and Webster [23] extend the model of Schweitzer and Cachon [12] by taking the shortage cost into consideration. They show that a loss-averse newsvendor may order more than a risk-neutral newsvendor when shortage cost is relatively high. Wang [24] further extends the loss-averse newsvendor problem to a more complex game setting where multiple newsvendors with loss-aversion preferences are competing for inventory from a risk-neutral supplier. These studies investigate loss aversion from the perspective of the newsvendor problem and fail to analyze the topic from the viewpoint of supply chain management. Wang and Webster [16] consider the channel coordination of a supply chain with a risk-neutral manufacturer and a loss-averse retailer. They investigate the role of a gain/loss sharing provision for mitigating the loss-aversion effect that decreases the retailer order quantity and total supply chain profit. They also present distribution-free GLB contracts that can achieve supply chain coordination and arbitrarily distribute the expected supply chain profit between the manufacturer and the retailer. Chen and Xiao [25] develop three (re)ordering models for a supply chain that consist of one risk-neutral manufacturer and one loss-averse retailer. They also design a buyback-setup cost-sharing mechanism to coordinate the supply chain for each policy. Chen et al. [15] study channel coordination with a loss-averse retailer that orders from a risk-neutral supplier via option contracts. This study considers sales-effort dependent demand to extend the model of Wang and Webster [16]. Our study focuses on identifying contracts that can coordinate the supply chain. We also discuss the effect of loss aversion on the ordering and effort policies of the retailer and on supply chain performance.

## 3. Model Development

We consider a supply chain in which a manufacturer sells a perishable product to a retailer. We assume that the manufacturer, as a large diversified firm, is risk neutral, and the retailer, as a smaller and less diversified firm, is loss averse. The retailer faces a stochastic demand that is sensitive to sales effort.

### 3.1. Notations and Assumptions

We introduce the notations that will be used in the formulation. Let *p* be the unit retail price of the retailer, *c* be the unit production cost of the manufacturer, *w* be the unit wholesale price of the manufacturer, *v* be the unit value of unsold products, and *Q* be the order quantity of the retailer (or the production quantity of the manufacturer in the integrated supply chain). We use *b* ∈ [*v*, *w*) as the buyback credit, *β* ∈ [0,1) as the manufacturer's sharing fraction of the retailer's gain, and *γ* ∈ [0,1) as the manufacturer's sharing fraction of the retailer's loss under settings with a GLB contract. We use *T* as the sales target and *τ* as the rebate (or penalty) under settings with a SRP contract. We use a single effort level *e* to summarize the activities of the retailer in promoting sales. Then, we let *g*(*e*) be the retailer's cost of exerting an effort level *e*, where *g*(0) = 0, *g*′(*e*) > 0, and *g*′′(*e*) > 0. Thus, marginal effort cost is increasing. Customer demand is modeled in a multiplicative form. In particular, let *X* = *φ*(*e*) · *ε* denote the demand, where *ε* is a positive stochastic variable. We assume that *φ*(*e*) is a concave and increasing function in an effort; that is, *φ*′(*e*) > 0 and *φ*′′(*e*) ≤ 0, which indicates that the marginal effectiveness of the effort is decreasing [5, 20]. Let *f*(*x*) be the probability density function (PDF) of demand *X* and let *F*(*x*) be the corresponding cumulative distribution function (CDF). Similar to most traditional newsvendor models, we assume that *F*(*x*) is differentiable, invertible, and strictly increasing over a nonnegative and continuous range. Let *f*(*x*∣*e*) be the PDF and let *F*(*x*∣*e*) be the CDF of demand, given the effort level *e*. In addition, we assume that demand is stochastically increasing in effort; that is, ∂*F*(*x*∣*e*)/∂*e* < 0.

Let *W*_0_ denote the reference level of the retailer (i.e., initial wealth) at the beginning of the selling season. We assume that gain (or loss) is perceived if the final wealth after the selling season is higher (or lower) than the initial wealth. We define the loss-aversion utility function of the retailer to be piecewise linear as follows [15, 16]:

(1) $$\begin{array}{c} U\left(W\right)=\{\begin{array}{cc} W-W_{0} & W\geq W_{0}\\ \lambda \left(W-W_{0}\right) & W<W_{0}, \end{array} \end{array}$$

where *λ* ≥ 1 is defined as the loss-aversion level and *W* is the final wealth of the retailer after the selling season. If *λ* = 1, then the retailer is risk neutral. If *λ* > 1, then a slope change occurs at the reference level, and higher values of *λ* imply higher levels of loss aversion. Without loss of generality, we normalize *W*_0_ = 0.

In addition, we assume that parameters *p*, *v*, and *λ* are known to both the retailer and the manufacturer. In case of shortages, unsatisfied demand carries no additional penalty. To avoid unrealistic and trivial cases, we assume that the following relationship is maintained: *p* > *w* > *c* > *v*.

### 3.2. Integrated Supply Chain

We start our analysis with the integrated supply chain. In this chain, a large and diversified risk-neutral manufacturer owns a retail channel and acts as the central planner of the supply chain. The objective of the central planner is to maximize the total expected supply chain profit by choosing effort level *e* and production quantity *Q*. The expected profit of the integrated supply chain is as follows:

(2) E[πT(Q,e)]=∫0Q[px+v(Q−x)]f(x ∣ e)dx +∫Q∞pQf(x ∣ e)dx−cQ−g(e).

After taking the first partial derivatives of (2) with respect to *Q* and *e*, respectively, we get

(3) $$\begin{array}{c} \frac{\partial E\left[\pi_{T}\left(Q,e\right)\right]}{\partial Q}=\left(p-c\right)-\left(p-v\right)F\left(Q\;|\;e\right), \end{array}$$

(4) $$\begin{array}{c} \frac{\partial E\left[\pi_{T}\left(Q,e\right)\right]}{\partial e}=-\left[\left(p-v\right)\overset{Q}{\underset{0}{\int }}\frac{\partial F\left(x\;|\;e\right)}{\partial e}dx+g^{\prime }\left(e\right)\right]. \end{array}$$

For a given *e*, it is easy to verify that *E*[*π*_*T*_(*Q*, *e*)] is concave in *Q*. Hence, the optimal production quantity *Q*_T_* should satisfy the following:

(5) $$\begin{array}{c} F\left(Q_{T}^{*}\;|\;e\right)=\frac{p-c}{p-v}. \end{array}$$

The following first-order conditions are necessary for coordination (but not necessarily sufficient):

(6) $$\begin{array}{c} \frac{\partial E\left[\pi_{T}\left(Q,e\right)\right]}{\partial Q}=\frac{\partial E\left[\pi_{T}\left(Q,e\right)\right]}{\partial e}=0. \end{array}$$

A contract designed by the manufacturer can coordinate the supply chain if it satisfies the first-order conditions at the optimal quantity-effort {*Q*_T_*, *e*_T_*}.

### 3.3. Decentralized Supply Chain

If a risk-neutral manufacturer and a loss-averse retailer are independent, they will attempt to maximize their individual expected profits without considering the expected total supply chain profit. In the decentralized decision system, the manufacturer acts as the leader and the retailer acts as the follower.

Based on the assumptions in Section 3.1, the expected profit function of the retailer under the wholesale price contract is

(7) E[πr(Q,e)]=∫0Q[px+v(Q−x)]f(x ∣ e)dx +∫Q∞pQf(x ∣ e)dx−wQ−g(e).

Let *q*(*Q*, *e*) = [(*w* − *v*)*Q* + *g*(*e*)]/(*p* − *v*) denote the breakeven selling quantity function of the retailer. If the realized demand *x* relative to *Q* is too low, that is, *x* < *q*(*Q*, *e*), then the retailer faces losses. If the realized demand is more than *q*(*Q*, *e*), then the retailer obtains gains. After mapping the retailer's expected profit function (7) into its utility function (1), we can express the expected utility of the retailer as follows:

(8) E[U(πr(Q,e))] =E[πr(Q,e)]+(λ−1)  ×∫0q(Q,e)[px+v(Q−x)−wQ−g(e)]f(x ∣ e)dx.

From (8), we find that the expected utility of the loss-averse retailer under the wholesale price contract is the sum of the expected profit and the expected loss that is biased by a factor of *λ* − 1. If *λ* = 1, then the retailer is risk neutral and the second term in (8) disappears.

After taking the first partial derivatives of (8) with respect to *Q* and *e*, respectively, we get

(9) ∂E[U(πr(Q,e))]∂Q =−(λ−1)(w−v)F(q(Q,e) ∣ e)  +p−w−(p−v)F(Q ∣ e),∂E[U(πr(Q,e))]∂e =(λ−1)  ×[(p−v)∫0q(Q,e)x∂f(x ∣ e)∂edx−g′(e)F(q(Q,e) ∣ e)]  −(p−v)∫0Q∂F(x ∣ e)∂edx−g′(e).

If the retailer is risk neutral (i.e., *λ* = 1), then the first terms in (9) disappear.

For a given *e*, the second partial derivative of *E*[*U*(*π*_*r*_(*Q*, *e*))] with respect to *Q* is

(10) ∂E2[U(πr(Q,e))]∂Q2 =−(λ−1)(w−v)2p−v  ×f(q(Q,e) ∣ e)−(p−v)f(Q ∣ e)<0.

Hence, *E*[*U*(*π*_*r*_(*Q*, *e*))] is concave in *Q* for a given *e*. Thus, the optimal order quantity of the retailer under the wholesale price contract, *Q*_λ_*, satisfies the following condition:

(11) −(λ−1)(w−v)F(q(Qλ∗,e) ∣ e)  +p−w−(p−v)F(Qλ∗ ∣ e)=0.

If the retailer is risk neutral, then it follows from (11) that the optimal order quantity *Q*_0_* of the retailer is unique and satisfies the following:

(12) $$\begin{array}{c} F\left(Q_{0}^{*}\;|\;e\right)=\frac{p-w}{p-v}. \end{array}$$

By comparing (5), (11), and (12), we can easily derive *Q*_λ_* < *Q*_0_* < *Q*_T_*, for *λ* > 1 and a given *e*. Therefore, for a given sales effort level, the optimal order quantity of the loss-averse retailer is less than that of either the risk-neutral retailer or the central planner of the integrated system. The jointly concavity of *E*[*U*(*π*_*r*_(*Q*, *e*))] in *Q* and *e* will be discussed in Section 5.

## 4. Coordinating Contracts

To encourage the loss-averse retailer to make additional orders, the risk-neutral manufacturer will offer an appropriate contract to the retailer for coordinating the supply chain. With supply chain coordination, the self-profit maximizing objective of the retailer is aligned with the objective of the whole supply chain during decision making. We use the solution of a risk-neutral integrated firm as the benchmark.

### 4.1. GLB Contracts

In this section, we investigate whether a distribution-free GLB contract can coordinate a supply chain with sales-effort dependent stochastic demand.

According to Wang and Webster [16], the GLB contract (*w*, *β*, *γ*, *b*) specifies that aside from paying the manufacturer the unit wholesale price *w*, the retailer receives *b* from the manufacturer for each unsold unit at the end of the selling season. Moreover, the manufacturer either shares a fraction *β* of the gain of the retailer or bears a fraction *γ* of its loss.

Let *q*_1_(*Q*, *e*) = [(*w* − *b*)*Q* + *g*(*e*)]/(*p* − *b*) denote the breakeven selling quantity function of the retailer under the GLB contract, and

(13) L1(Q,e) =∫0q1(Q,e)[px+b(Q−x)−wQ−g(e)]f(x ∣ e)dx

denote the expected loss function of the retailer. Moreover, let

(14) G1(Q,e)=∫q1(Q,e)Q[px+b(Q−x)−wQ−g(e)]f(x ∣ e)dx +∫Q∞[(p−w)Q−g(e)]f(x ∣ e)dx

denote the expected gain function of the retailer. Thus, the expected utility of the retailer under the GLB contract is

(15) E[U(πr(Q,e,β,γ,b))]=(1−β)E[πr(Q,e,b)] +[λ(1−γ)−(1−β)]L1(Q,e),

and the expected profit of the retailer is

(16) E[πr(Q,e,β,γ,b)]=(1−β)E[πr(Q,e,b)] +(β−γ)L1(Q,e),

where

(17) $$\begin{array}{c} E\left[\pi_{r}\left(Q,e,b\right)\right]=L_{1}\left(Q,e\right)+G_{1}\left(Q,e\right) \end{array}$$

is the expected profit of the retailer under the buyback contract.

Under the GLB contract, the expected profit function of the manufacturer is

(18) E[πm(Q,e,β,γ,b)] =(w−c)Q+γL1(Q,e)+βG1(Q,e)  −∫oQ(b−v)(Q−x)f(x ∣ e)dx.

From (2), (16), and (18), we obtain *E*[*π*_*T*_(*Q*, *e*)] = *E*[*π*_*r*_(*Q*, *e*, *β*, *γ*, *b*)]  +  *E*[*π*_*m*_(*Q*, *e*, *β*, *γ*, *b*)].

After taking the first partial derivatives of (15) with respect to *Q* and *e*, respectively, we have

(19) ∂E[U(πr(Q,e,β,γ,b))]∂Q =−[λ(1−γ)−(1−β)](w−b)F(q1(Q,e) ∣ e)  +(1−β)[p−w−(p−b)F(Q ∣ e)],∂E[U(πr(Q,e,β,γ,b))]∂e=[λ(1−γ)−(1−β)] ×[(p−b)∫0q1(Q,e)x∂f(x ∣ e)∂edx−g′(e)F(q1(Q,e) ∣ e)] −(1−β)[(p−b)∫0Q∂F(x ∣ e)∂edx+g′(e)].

**Proposition 1 .**

The following GLB contracts cannot coordinate the supply chain with sales-effort dependent demand:

(20) $$\begin{array}{c} \gamma =1-\frac{1-\beta }{\lambda },\\ b_{1}^{*}=b_{0}=p-\frac{\left(p-v\right)\left(p-w\right)}{\left(p-c\right)},\;b_{1}^{*}\neq v, \end{array}$$

where *b*_0_ = *p* − (*p* − *v*)(*p* − *w*)/(*p* − *c*) denotes the coordinating buyback credit for the risk-neutral retailer facing a stochastic demand that is not influenced by sales effort.

**Proof**

See Appendix.

Wang and Webster [16] demonstrate that GLB contracts with the parameters presented in (20) can coordinate the supply chain when sales effort does not influence demand. However, Proposition 1 shows that these contracts cannot achieve supply chain coordination with sales-effort dependent demand. From (20), we find that *w* = *c* if *b*_1_* = *v*. Note that the GLB contract reduces to a GL contract with three parameters (*β*, *γ*, *w*) if *b* = *v* and *w* = *c*. From the proof of Proposition 1, we obtain the following proposition.

**Proposition 2 .**

A GL contract with *γ* = 1 − (1 − *β*)/*λ* and *w* = *c* can coordinate the supply chain and achieve an arbitrary allocation of the optimal expected supply chain profit between the manufacturer and the retailer by changing the value of *β*.

**Proof**

See Appendix.

Proposition 2 identifies a special class of coordinating GL contracts wherein the gain-sharing and loss-sharing fractions satisfy *γ* = 1 − (1 − *β*)/*λ*, and the wholesale price satisfies *w* = *c*. Under this contract, the wholesale price eliminates double marginalization, the loss-sharing fraction eliminates the loss-aversion effect, and the gain-sharing fraction influences the allocation of the expected profits between the two firms.

### 4.2. GLB Contract Combined with SRP

Under a SRP contract, the manufacturer sets up a product sales target *T* for the retailer. If retail sales exceeds the target, then the manufacturer will give the retailer a rebate, that is, a reward of *τ* for each unit of product sold above *T*. Otherwise, the retailer will need to pay the manufacturer a penalty, that is, a payment of *τ* for each unit of unsold product below *T*. Next, we investigate the role of the GLB contract combined with SRP for supply chain coordination.

We define *q*_2_(*Q*, *e*) = [(*w* − *b*)*Q* + *g*(*e*) + *τT*]/(*p* − *b* + *τ*) as the breakeven selling quantity function of the retailer under the GLB contract combined with SRP,

(21) L2(Q,e)=∫0q2(Q,e)[px+b(Q−x)−wQ−g(e)+τ(x−T)]f(x ∣ e)dx

as the expected loss function of the retailer, and

(22) G2(Q,e)=∫q2(Q,e)Q[px+b(Q−x)−wQ−g(e)+τ(x−T)]×f(x ∣ e)dx +∫Q∞[(p−w)Q−g(e)+τ(Q−T)]×f(x ∣ e)dx

as the expected gain function of the retailer. Then, the expected utility of the retailer under the GLB contract combined with SRP is

(23) E[U(πr(Q,e,β,γ,b,τ))]=(1−β)E[πr(Q,e,b,τ)] +[λ(1−γ)−(1−β)]L2(Q,e),

and the expected profit of the retailer is

(24) E[πr(Q,e,β,γ,b,τ)]=(1−β)E[πr(Q,e,b,τ)] +(β−γ)L2(Q,e),

where

(25) $$\begin{array}{c} E\left[\pi_{r}\left(Q,e,b,\tau \right)\right]=L_{2}\left(Q,e\right)+G_{2}\left(Q,e\right) \end{array}$$

is the expected profit of the retailer under the buyback contract combined with SRP.

Under the GLB contract combined with SRP, the expected profit function of the manufacturer can be written as

(26) E[πm(Q,e,β,γ,b,τ)]=(w−c)Q+γL2(Q,e)+βG2(Q,e) −∫oQ(b−v)(Q−x)f(x ∣ e)dx −∫0Qτ(x−T)f(x ∣ e)dx −∫Q∞τ(Q−T)f(x ∣ e)dx.

From (2), (24), and (26), we obtain *E*[*π*_*T*_(*Q*, *e*)] = *E*[*π*_*r*_(*Q*, *e*, *β*, *γ*, *b*, *τ*)]   +  *E*[*π*_*m*_(*Q*, *e*, *β*, *γ*, *b*, *τ*)].

After taking the first partial derivatives of (23) with respect to *Q* and *e*, respectively, we get

(27) ∂E[U(πr(Q,e,β,γ,b,τ))]∂Q =−[λ(1−γ)−(1−β)](w−b)F(q2(Q,e) ∣ e)  +(1−β)[p−w+τ−(p−b+τ)F(Q ∣ e)],∂E[U(πr(Q,e,β,γ,b,τ))]∂e=[λ(1−γ)−(1−β)] ×[(p−b+τ)×∫0q2(Q,e)x∂f(x ∣ e)∂edx−g′(e)F(q2(Q,e) ∣ e)] −(1−β)[(p−b+τ)∫0Q∂F(x ∣ e)∂edx+g′(e)].

**Proposition 3 .**

The following GLB contract combined with SRP can coordinate the supply chain with sales-effort dependent demand:

(28) $$\begin{array}{c} \gamma =1-\frac{1-\beta }{\lambda },\;\;b_{2}^{*}=w+v-c,\\ \tau^{*}=b_{2}^{*}-v. \end{array}$$

**Proof**

See Appendix.

Under the GLB contract combined with SRP and the contract parameters presented in (28), the expected profit of the retailer (24) can be written as follows:

(29) E[πr(QT∗,eT∗,β,γ,b2∗,τ∗)] =(1−β){(−λ−1λ)L2(QT∗,eT∗)+E[πr(QT∗,eT∗,b2∗,τ∗)]}.

From (29), we can see that the expected profit of the retailer is decreasing in *β*. Thus, the expected profit of the manufacturer is increasing in *β*. Under the GLB contract combined with SRP, buyback credit and SRP policy eliminate double marginalization, the loss-sharing fraction eliminates the loss-aversion effect, and the gain-sharing fraction influences the allocation of the expected supply chain profit between the two firms.

**Proposition 4 .**

Under the GLB contract combined with SRP and the contract parameters presented in (28), an arbitrary allocation of the optimal expected supply chain profit between the manufacturer and the retailer can be achieved by changing the value of *T*.

**Proof**

See Appendix.

As Cachon [7] points out, if a contract can arbitrarily allocate supply chain profit, then there always exists a coordinating contract under which each firm's profit is no worse off and at least one firm is strictly better off.  Proposition 4 shows that the GLB contract combined with SRP is able to lead to a Pareto improving win-win situation for supply chain members.

## 5. Efficiency of Coordination

In this section, we first examine some properties that concern the optimal solution (*Q*_λ_*, *e*_λ_*) in the decentralized system and then investigate coordination efficiency.

To facilitate further analysis, we let *X*(*Q*, *e*) and *Y*(*Q*, *e*) denote the first partial derivatives of (8) with respect to *Q* and *e*, respectively. As

(30) $$\begin{array}{c} f\left(x\;|\;e\right)=\frac{1}{\varphi \left(e\right)}f\left(\frac{x}{\varphi \left(e\right)}\right),\;\;F\left(x\;|\;e\right)=F\left(\frac{x}{\varphi \left(e\right)}\right), \end{array}$$

we can rewrite (9) as follows:

(31) X(Q,e)=∂E[U(πr(Q,e))]∂Q=−(λ−1)(w−v)F(q(Q,e)φ(e)) +p−w−(p−v)F(Qφ(e)),

(32) Y(Q,e) =∂E[U(πr(Q,e))]∂e =(λ−1)  ×[(p−v)φ′(e)×∫0q(Q,e)/φ(e)xf(x)dx−g′(e)F(q(Q,e)φ(e))] +(p−v)φ′(e)∫0Q/φ(e)xf(x)dx−g′(e).

**Proposition 5 .**

The retailer's expected utility function, *E*[*U*(*π*_*r*_(*Q*, *e*))], is jointly concave in *Q* and *e*.

**Proof**

See Appendix.

Since *E*[*U*(*π*_*r*_(*Q*, *e*))] is jointly concave in *Q* and *e*, the optimal solution (*Q*_λ_*, *e*_λ_*) can be obtained through the first-order conditions.

Next, we examine the effects of loss aversion on the optimal sales effort and order quantity of the retailer.

**Property 1 .**

For any *λ* ≥ 1, *e*_λ_* is decreasing in *λ*.

**Proof**

See Appendix.

**Property 2 .**

*Q*_λ_* is increasing in *e*_λ_* when *λ* → 1 or (*Q*_*λ*_, *e*_*λ*_) satisfy

(33) $$\begin{array}{c} \left(w-v\right)Q_{\lambda }\varphi^{\prime }\left(e_{\lambda }\right)-\left[g^{\prime }\left(e_{\lambda }\right)\varphi \left(e_{\lambda }\right)-g\left(e_{\lambda }\right)\varphi^{\prime }\left(e_{\lambda }\right)\right]>0. \end{array}$$

**Proof**

See Appendix.

**Property 3 .**

*Q*_λ_* is decreasing in *λ* when *λ* → 1 or (33) holds.

**Proof**

See Appendix.

Property 1 shows that the optimal effort of the retailer decreases as loss aversion increases.  Property 3 shows that, with the increasing of loss aversion, the optimal order quantity of the retailer decreases when loss-aversion level tends toward 1, or when the sales effort and order quantity of the retailer satisfy a certain relationship.

From Properties 1 and 3, we can easily derive the following proposition, and hence the proof is omitted.

**Proposition 6 .**

For any *λ* > 1, *e*_λ_* < *e*_0_*; *Q*_λ_* < *Q*_0_* when *λ* → 1 or (33) holds.

From Proposition 6, we can see that the optimal effort *e*_λ_* of the loss-averse retailer is always less than the optimal effort *e*_0_* of the risk-neutral retailer. In addition, the optimal order quantity *Q*_λ_* of the loss-averse retailer is less than the optimal order quantity *Q*_0_* of the risk-neutral retailer when *λ* → 1 or (33) holds.

**Lemma 7 .**

Consider *e*_0_* < *e*_T_*, *Q*_0_* < *Q*_T_*.

**Proof**

See Appendix.

Lemma 7 shows that the optimal effort *e*_0_* of the risk-neutral retailer is less than the optimal effort *e*_T_* of the integrated supply chain. Moreover, the optimal order quantity *Q*_0_* of the risk-neutral retailer is less than the optimal production quantity *Q*_T_* of the integrated supply chain.

From Proposition 6 and Lemma 7, we can easily derive the following results, and hence, the proof is omitted.

**Proposition 8 .**

For any *λ* > 1, *e*_λ_* < *e*_T_*; *Q*_λ_* < *Q*_T_* if *λ* → 1 or (33) holds.

Proposition 8 shows that the optimal effort of the loss-averse retailer is always less than that of the integrated supply chain. Moreover, the optimal order quantity of the loss-averse retailer is less than that of the integrated supply chain when *λ* → 1 or (33) holds. As a consequence, the expected profit of the decentralized supply chain with a loss-averse retailer is lower than that of the integrated supply chain.

## 6. Numerical Example

We conduct a numerical study to illustrate the model and gain additional insights. First, we specify that *φ*(*e*) = *ae* and *g*(*e*) = *μe* ^2^/2, where *a* > 0, which denotes the influence degree of the effort level on the expected demand, and *μ* > 0, which denotes the costliness of effort. The base parameters of the model are as follows: *p* = 10, *w* = 8, *c* = 4, *v* = 0, *a* = 400, *μ* = 4, and *λ* = 2. Random variable *ε* is assumed to be uniformly distributed with support [0, 1].

Table 1 exhibits the differences in equilibrium strategies of the integrated and decentralized systems. From Table 1, one can easily find that the optimal effort level and order quantity in the decentralized system are lower than those in the integrated system. As a consequence, the expected profit of the decentralized system is lower than that of the integrated system. In addition, we define the percentage, Δ, by which the expected profit of the integrated system increases over the expected profit of the decentralized system as

(34) $$\begin{array}{c} \Delta =\frac{E\left[\pi_{s}^{T}\left(Q_{T}^{*},e_{T}^{*}\right)\right]-E\left[\pi_{s}^{d}\left(Q_{\lambda }^{*},e_{\lambda }^{*}\right)\right]}{E\left[\pi_{s}^{d}\left(Q_{\lambda }^{*},e_{\lambda }^{*}\right)\right]}. \end{array}$$

From Table 1, one can easily see that Δ = 2381.8%, suggesting that the value of coordination can be significant.

Next, we investigate the influence of the gain-sharing fraction and the sales-target level on the allocation of the expected supply chain profit after coordination is achieved between the manufacturer and the retailer. The results are provided in Tables 2 and 3.  Table 2 indicates that, under the coordinating GL contract, the expected profit of the retailer is decreasing in *β*, whereas the expected profit of the manufacturer is increasing in *β*. Through computations, we find that when 0.0638 ≤ *β* ≤ 0.9926, *E*[*π*_*r*_^*T*^(*Q*_T_*, *e*_T_*)] ≥ *E*[*π*_*r*_^*d*^(*Q*_λ_*, *e*_λ_*)] and *E*[*π*_*m*_^*T*^(*Q*_T_*, *e*_T_*)] ≥ *E*[*π*_*m*_^*d*^(*Q*_λ_*, *e*_λ_*)], where *E*[*π*_*r*_^*T*^(*Q*_T_*, *e*_T_*)] and *E*[*π*_*m*_^*T*^(*Q*_T_*, *e*_T_*)] represent the profits of the retailer and the manufacturer in the coordinated supply chain, respectively, and *E*[*π*_*r*_^*d*^(*Q*_λ_*, *e*_λ_*)] and *E*[*π*_*m*_^*d*^(*Q*_λ_*, *e*_λ_*)] represent the profits of the retailer and the manufacturer in the decentralized supply chain, respectively. Thus, by choosing the proper value of *β*, the coordinated supply chain will reach Pareto improving.  Table 3 indicates that under the coordinating GLB contract combined with SRP, the expected profit of the retailer is decreasing in *T*, whereas the expected profit of the manufacturer is increasing in *T*. We find that the coordinating GLB contract combined with SRP at a fixed *β*_0_ = 0.0322 can arbitrarily allocate the optimal expected supply chain profit between the manufacturer and retailer by changing the value of *T*. Moreover, *E*[*π*_*r*_^*T*^(*Q*_T_*, *e*_T_*)] ≥ *E*[*π*_*r*_^*d*^(*Q*_λ_*, *e*_λ_*)] and *E*[*π*_*m*_^*T*^(*Q*_T_*, *e*_T_*)] ≥ *E*[*π*_*m*_^*d*^(*Q*_λ_*, *e*_λ_*)] when 562 ≤ *T* ≤ 17229. Hence, the coordinated supply chain will reach Pareto improving by choosing the proper value of *T*.

Table 4 shows the effect of the loss-aversion level on the optimal-effort level, order quantity, and the corresponding expected profit of the decentralized system. The values of the other parameters in Table 4 are maintained the same as the base parameters. Table 4 shows that the optimal effort level, order quantity, and the expected profit of the decentralized system decrease as loss-aversion level *λ* increases. These results are consistent with those presented in Properties 1 and 3, which further verifies these properties numerically.

In what follows, we explore the effects of changes in parameters *a* and *μ* on the optimal effort level, order/production quantity, and the resulting expected profit of the system after coordination. The results are shown in Tables 5 and 6. The values of the other parameters in the two tables are maintained the same as the base parameters.  Table 5 indicates that as the influence degree of the effort level on demand increases; the optimal effort level, order/production quantity, and the resulting expected profit of the system after coordination also increase.  Table 6 shows that the optimal effort level, order/production quantity, and the resulting expected profit of the system after coordination decrease as the costliness of effort increases.

## 7. Conclusions

We consider a single-period, two-echelon supply chain composed of a risk-neutral manufacturer and a loss-averse retailer facing stochastic demand that is sensitive to sales effort. Under the loss-averse newsvendor setting, the distribution-free GLB contract has been shown to be able to coordinate the supply chain. However, we show that the GLB contract remains ineffective in managing the supply chain when retailer sales effort influences demand. To effectively coordinate the channel, we propose a GLB contract combined with SRP. Under this contract, the buyback credit and SRP policy eliminate double marginalization, the loss-sharing fraction eliminates the loss-aversion effect, and the gain-sharing fraction influences the allocation of expected supply chain profit between the two firms. Therefore, the GLB contract combined with SRP can coordinate the supply chain and achieve Pareto improvement. In addition, we discover a special class of GL contracts that can coordinate the supply chain and arbitrarily allocate the expected supply chain profit between the manufacturer and the retailer.

Subsequently, we analyze the effect of loss aversion on the decision-making behavior of the retailer and supply chain performance. We find that the optimal effort of the retailer decreases as loss aversion increases. In addition, we show that with the increasing of loss aversion, the optimal order quantity of the retailer decreases when the loss-aversion level tends toward 1, or when the sales effort and order quantity of the retailer satisfy a certain relationship. Through numerical studies, we further confirm that coordination can significantly improve supply chain performance. We also find that under the coordinating GL contract, the supply chain will reach Pareto improving by choosing the proper gain-sharing fraction. Meanwhile, under the coordinating GLB contract combined with SRP, the supply chain will reach Pareto improving by choosing the proper sales target for the retailer at a fixed gain-sharing fraction.

As an extension of this work, future research can consider a supply chain that comprises a risk-neutral manufacturer and multiple loss-averse retailers, as well as adding competitive characteristics to the model. In addition, future research can adopt other nonlinear utility functions that can better describe the decision-making behavior of the retailer.

## Figures and Tables

**Table 1:** Optimal results of the integrated and decentralized systems.

Model type	Effort level	Order quantity	Retailer's profit	Manufacturer's profit	System profit	Δ (% profit increase)
Decentralized	11.1	529	496	2115	2611	2381.8
Integrated	180.0	43200	26782	38018	64800	—

**Table 2:** Effect of β on the allocation of the expected supply chain profit between two firms.

β	0.0	0.2	0.4	0.6	0.8	1.0
*E*[π_*r*_^*T*^(*Q*_T_*, *e*_T_*)]	66956	53565	40174	26782	13391	0
*E*[π_*m*_^*T*^(*Q*_T_*, *e*_T_*)]	−2156	11235	24626	38018	51409	64800

**Table 3:** Effect of *T* on the allocation of the expected supply chain profit between two firms.

*T*	0	3000	6000	9000	12000	15000	18000
*E*[π_*r*_^*T*^(*Q*_T_*, *e*_T_*)]	64800	53519	42271	31059	19883	8746	−2351
*E*[π_*m*_^*T*^(*Q*_T_*, *e*_T_*)]	0	11281	22529	33741	44917	56054	67151

**Table 4:** Effect of λ on the optimal policies of the decentralized system.

λ	*e*_λ_*	*Q*_λ_*	*E*[π_*s*_^*d*^(*Q*_λ_*, *e*_λ_*)]
2	11.1	529	2612
3	7.7	261	1337
4	5.9	155	811
5	4.8	103	544

**Table 5:** Effect of *a* on the optimal policies of the system after coordination.

*a*	*e*_T_*	*Q*_T_*	*E*[π_*s*_^*T*^(*Q*_T_*, *e*_T_*)]
50	22.5	675	1013
100	45	2700	4050
200	90	10800	16200
400	180	43200	64800

**Table 6:** Effect of μ on the optimal policies of the system after coordination.

μ	*e*_T_*	*Q*_T_*	*E*[π_*s*_^*T*^(*Q*_T_*, *e*_T_*)]
1	720	172800	259200
2	360	86400	129600
4	180	43200	64800
8	90	21600	32400

## Appendix

Proof of Proposition 1. If the GLB contract parameters satisfy (20), from (19), we get

(A.1) ∂E[U(πr(Q,e,β,γ,b1∗))]∂Q =(1−β)(p−w)p−c[p−c−(p−v)F(Q ∣ e)],

(A.2) ∂E[U(πr(Q,e,β,γ,b1∗))]∂e =−(1−β)[(p−v)(p−w)p−c∫0Q∂F(x ∣ e)∂edx+g′(e)].

By comparing (A.1) with (3), we find that *Q*_*T*_* satisfies ∂*E*[*U*(*π*_*r*_(*Q*_*T*_*, *e*, *β*, *γ*, *b*_1_*))]/∂*Q* = 0. However, by comparing (A.2) with (4), we find that *e*_*T*_* can never satisfy ∂*E*[*U*(*π*_*r*_(*Q*, *e*_*T*_*, *β*, *γ*, *b*_1_*))]/∂*e* = 0 given *b*_1_* ≠ *v*. Hence, when sales effort influences demand, the supply chain cannot be coordinated through a GLB contract with the parameters presented in (20).

Proof of Proposition 2. From (A.1) and (A.2), we can see that if *b* = *v* and *w* = *c*, then *Q*_*T*_* and *e*_*T*_* satisfy

(A.3) $$\begin{array}{c} \frac{\partial E\left[U\left(\pi_{r}\left(Q_{T}^{*},e,\beta ,\gamma ,v\right)\right)\right]}{\partial Q}=0,\\ \frac{\partial E\left[U\left(\pi_{r}\left(Q,e_{T}^{*},\beta ,\gamma ,v\right)\right)\right]}{\partial e}=0. \end{array}$$

Therefore, a GL contract with *γ* = 1 − (1 − *β*)/*λ* and *w* = *c* can coordinate the supply chain. Under the GL contract with *γ* = 1 − (1 − *β*)/*λ* and *w* = *c*, *E*[*π*_*r*_(*Q*_*T*_*, *e*_*T*_*, *v*)] = *E*[*π*_*T*_(*Q*_*T*_*, *e*_*T*_*)]. Therefore, the retailer's expected profit function (16) can be rewritten as

(A.4) E[πr(QT∗,eT∗,β,γ,v)] =(1−β){[−λ−1λ]L1(QT∗,eT∗)+E[πT(QT∗,eT∗)]}.

From (A.4), we can see that if *β* = 0, then *E*[*π*_*r*_(*Q*_*T*_*, *e*_*T*_*, *β*, *γ*, *v*)] > *E*[*π*_*T*_(*Q*_*T*_*, *e*_*T*_*)]; if *β* = 1, then *E*[*π*_*r*_(*Q*_*T*_*, *e*_*T*_*, *β*, *γ*, *v*)] = 0. Because *E*[*π*_*r*_(*Q*_*T*_*, *e*_*T*_*, *β*, *γ*, *v*)] is continuous in *β*, the coordinating GL contract can achieve an arbitrary allocation of the optimal expected supply chain profit between the manufacturer and the retailer by changing the value of *β*.

Proof of Proposition 3. If the parameters of the GLB contract combined with SRP satisfy (28), then we can rewrite (27) as follows:

(A.5) ∂E[U(πr(Q,e,β,γ,b2∗,τ∗))]∂Q =(1−β)[p−c−(p−v)F(Q ∣ e)],

(A.6) ∂E[U(πr(Q,e,β,γ,b2∗,τ∗))]∂e =−(1−β)[(p−v)∫0Q∂F(x ∣ e)∂edx+g′(e)].

By comparing (A.5) with (3), we find that *Q*_*T*_* satisfies ∂*E*[*U*(*π*_*r*_(*Q*_*T*_*, *e*, *β*, *γ*, *b*_2_*, *τ**))]/∂*Q* = 0. By comparing (A.6) with (4), we find that *e*_*T*_* satisfies ∂*E*[*U*(*π*_*r*_(*Q*, *e*_*T*_*, *β*, *γ*, *b*_2_*, *τ**))]/∂*e* = 0. Thus, the GLB contract combined with SRP and the contract parameters presented in (28) can coordinate the supply chain.

Proof of Proposition 4. Under the GLB contract combined with SRP and the contract parameters presented in (28), we obtain *E*[*π*_*r*_(*Q*_*T*_*, *e*_*T*_*, *b*_2_*, *τ**)] = *E*[*π*_*T*_(*Q*_*T*_*, *e*_*T*_*)]−(*w* − *c*)*T* from (25). Therefore, the retailer's expected profit (24) can be rewritten as

(A.7) E[πr(QT∗,eT∗,β,γ,b2∗,τ∗)] =(1−β){[−λ−1λ]L2(QT∗,eT∗)−(w−c)T+E[πT(QT∗,eT∗)]}.

If *T* = 0, from (A.7), we can see that if *β* = 0, then *E*[*π*_*r*_(*Q*_*T*_*, *e*_*T*_*, *β*, *γ*, *b*_2_*, *τ**)] > *E*[*π*_*T*_(*Q*_*T*_*, *e*_*T*_*)]; if *β* = 1, then *E*[*π*_*r*_(*Q*_*T*_*, *e*_*T*_*, *β*, *γ*, *b*_2_*, *τ**)] = 0. Therefore, we can always find a *β* = *β*_0_ ∈ (0,1) at which *E*[*π*_*r*_(*Q*_*T*_*, *e*_*T*_*, *β*, *γ*, *b*_2_*, *τ**)] = *E*[*π*_*T*_(*Q*_*T*_*, *e*_*T*_*)]. Similarly, from (A.7), we can always find a *T* = *T*_0_ at which *E*[*π*_*r*_(*Q*_*T*_*, *e*_*T*_*, *β*, *γ*, *b*_2_*, *τ**)] = 0. Since *E*[*π*_*r*_(*Q*_*T*_*, *e*_*T*_*, *β*, *γ*, *b*_2_*, *τ**)] is continuous in *T*, the coordinating GLB contract combined with SRP at a fixed *β* = *β*_0_ can arbitrarily allocate the expected supply chain profit between the manufacturer and the retailer by changing the value of *T* ∈ [0, *T*_0_].

Proof of Proposition 5. The Hessian matrix of the expected utility function of the retailer is

(A.8) $$\begin{array}{c} H_{E[U]}=\left[\begin{array}{cc} \frac{\partial^{2}E\left[U\left(\pi_{r}\left(Q,e\right)\right)\right]}{\partial Q^{2}} & \frac{\partial^{2}E\left[U\left(\pi_{r}\left(Q,e\right)\right)\right]}{\partial Q\partial e}\\ \frac{\partial^{2}E\left[U\left(\pi_{r}\left(Q,e\right)\right)\right]}{\partial e\partial Q} & \frac{\partial^{2}E\left[U\left(\pi_{r}\left(Q,e\right)\right)\right]}{\partial e^{2}} \end{array}\right]. \end{array}$$

Denote

(A.9) $$\begin{array}{c} \frac{\partial^{2}E\left[U\left(\pi_{r}\left(Q,e\right)\right)\right]}{\partial Q^{2}}=A+B,\\ \frac{\partial^{2}E\left[U\left(\pi_{r}\left(Q,e\right)\right)\right]}{\partial Q\partial e}=C+D,\\ \frac{\partial^{2}E\left[U\left(\pi_{r}\left(Q,e\right)\right)\right]}{\partial e^{2}}=E+F+G+H+I+J. \end{array}$$

From (31) and (32), we obtain

(A.10) A=−p−vφ(e)f(Qφ(e)),B=−(λ−1)(w−v)2(p−v)φ(e)f(q(Q,e)φ(e)),C=(p−v)Qφ′(e)φ2(e)f(Qφ(e)),D=(λ−1) ×(w−v)[(p−v)q(Q,e)φ′(e)−g′(e)φ(e)](p−v)φ2(e) ×f(q(Q,e)φ(e)),E=(p−v)φ′′(e)∫0Q/φ(e)xf(x)dx,F=−(p−v)(Qφ′(e))2φ3(e)f(Qφ(e)),G=−g′′(e),H=(λ−1)(p−v)φ′′(e)∫0q(Q,e)/φ(e)xf(x)dx,I=−(λ−1) ×[(p−v)q(Q,e)φ′(e)−g′(e)φ(e)]2(p−v)φ3(e) ×f(q(Q,e)φ(e)),J=−(λ−1)g′′(e)F(q(Q,e)φ(e)).

Note that *AF* = *C* ^2^, *BI* = *D* ^2^, and *AI* + *BF* − 2*CD* > 0. The assumptions ensure that ∂^2^ *E*[*U*(*π*_*r*_(*Q*, *e*))]/∂*Q*∂*e* = ∂^2^ *E*[*U*(*π*_*r*_(*Q*, *e*))]/∂*e*∂*Q*. Define |*H*_*E*(*U*)_| as the Hessian matrix determinant; then from (A.8) we get

(A.11) |HE(U)|=(A+B)(E+F+G+H+I+J)−(C+D)2>(A+B)(E+H)+(A+B)(G+J).

Note that *A* + *B* < 0, *G* + *J* < 0, and *E* + *H* < 0. Thus, we get |*H*_*E*(*U*)_| > 0. Therefore, the Hessian matrix of the expected utility function of the retailer is negative definite, which implies that *E*[*U*(*π*_*r*_(*Q*, *e*))] is jointly concave in *Q* and *e*.

Proof By the implicit function theorem, from (31) and (32), we have

(A.12) $$\begin{array}{c} \frac{de_{\lambda }^{*}}{d\lambda }=-\frac{\partial \left(X,Y\right)}{\partial \left(Q_{\lambda }^{*},\lambda \right)}\cdot \frac{1}{{\left|H_{E[U]}\right|}_{\left(Q_{\lambda }^{*},e_{\lambda }^{*}\right)}}, \end{array}$$

where

(A.13) $$\begin{array}{c} \frac{\partial \left(X,Y\right)}{\partial \left(Q_{\lambda }^{*},\lambda \right)}=\left|\begin{array}{cc} \frac{\partial^{2}E\left[U\left(\pi_{r}\left(Q_{\lambda }^{*},e_{\lambda }^{*}\right)\right)\right]}{\partial Q^{2}} & \frac{\partial^{2}E\left[U\left(\pi_{r}\left(Q_{\lambda }^{*},e_{\lambda }^{*}\right)\right)\right]}{\partial Q\partial \lambda }\\ \frac{\partial^{2}E\left[U\left(\pi_{r}\left(Q_{\lambda }^{*},e_{\lambda }^{*}\right)\right)\right]}{\partial e\partial Q} & \frac{\partial^{2}E\left[U\left(\pi_{r}\left(Q_{\lambda }^{*},e_{\lambda }^{*}\right)\right)\right]}{\partial e\partial \lambda } \end{array}\right|. \end{array}$$

Denote

(A.14) ∂2E[U(πr(Qλ∗,eλ∗))]∂Q∂λ=−(w−v)F(q(Qλ∗,eλ∗)φ(eλ∗))=M,∂2E[U(πr(Qλ∗,eλ∗))]∂e∂λ=(p−v)φ′(eλ∗) ×∫0q(Qλ∗,eλ∗)/φ(eλ∗)xf(x)dx−g′(eλ∗)F(q(Qλ∗,eλ∗)φ(eλ∗))=N.

It is easy to verify that *N* ≤ *W*, where

(A.15) W=(p−v)q(Qλ∗,eλ∗)φ′(eλ∗)−g′(eλ∗)φ(eλ∗)φ(eλ∗) ×F(q(Qλ∗,eλ∗)φ(eλ∗)).

From Proof of Proposition 5, *A* + *B* < 0. Hence,

(A.16) ∂2E[U(πr(Qλ∗,eλ∗))]∂Q2·∂2E[U(πr(Qλ∗,eλ∗))]∂e∂λ =(A+B)N≥(A+B)W.

It is noted that

(A.17) $$\begin{array}{c} \frac{\partial^{2}E\left[U\left(\pi_{r}\left(Q_{\lambda }^{*},e_{\lambda }^{*}\right)\right)\right]}{\partial Q\partial \lambda }\cdot \frac{\partial^{2}E\left[U\left(\pi_{r}\left(Q_{\lambda }^{*},e_{\lambda }^{*}\right)\right)\right]}{\partial e\partial Q}=\left(C+D\right)M. \end{array}$$

From (A.13), (A.16), and (A.17), we obtain

(A.18) ∂(X,Y)∂(Qλ∗,λ)=(A+B)N−(C+D)M≥(A+B)W−(C+D)M.

It is easy to verify that *BW* − *DM* = 0, and

(A.19) AW−CM =p−vφ(eλ∗)f(Qλ∗φ(eλ∗)) ·g′(eλ∗)φ(eλ∗)−(p−v)q(Qλ∗,eλ∗)φ′(eλ∗)φ(eλ∗) ×F(q(Qλ∗,eλ∗)φ(eλ∗))+(p−v)Qλ∗φ′(eλ∗)φ2(eλ∗) ×f(Qλ∗φ(eλ∗))·(w−v)F(q(Qλ∗,eλ∗)φ(eλ∗)) =(p−v)φ(eλ∗)g′(eλ∗)−φ′(eλ∗)g(eλ∗)φ2(eλ∗) ×f(Qλ∗φ(eλ∗))F(q(Qλ∗,eλ∗)φ(eλ∗)).

Based on our assumptions, it is easy to show that *φ*(*e*)*g*′(*e*) − *φ*′(*e*)*g*(*e*) > 0 for any *e*. From (A.19), we obtain *AW* − *CM* > 0. Therefore, ∂(*X*, *Y*)/∂(*Q*_*λ*_*, *λ*) > 0. From the Proof of Proposition 5, |*H*_*E*(*U*)_| > 0. After combining the above results, we get *de*_*λ*_*/*dλ* < 0.

Proof By the implicit function theorem, from (31), we get

(A.20) $$\begin{array}{c} \frac{\partial Q_{\lambda }^{*}}{\partial e_{\lambda }^{*}}=-\frac{\partial X\left(Q_{\lambda }^{*},e_{\lambda }^{*}\right)/\partial e}{\partial X\left(Q_{\lambda }^{*},e_{\lambda }^{*}\right)/\partial Q}=-\frac{\partial E^{2}\left[U\left(\pi_{r}\left(Q_{\lambda }^{*},e_{\lambda }^{*}\right)\right)\right]/\partial Q\partial e}{\partial E^{2}\left[U\left(\pi_{r}\left(Q_{\lambda }^{*},e_{\lambda }^{*}\right)\right)\right]/\partial Q^{2}}. \end{array}$$

From Proof of Proposition 5, ∂*E* ^2^[*U*(*π*_*r*_(*Q*_*λ*_*, *e*_*λ*_*))]/∂*Q* ^2^  <  0, and if (*w* − *v*)*Q*_*λ*_ *φ*′(*e*_*λ*_)−[*g*′(*e*_*λ*_)*φ*(*e*_*λ*_) − *g*(*e*_*λ*_)*φ*′(*e*_*λ*_)] > 0 or *λ* → 1, then ∂*E* ^2^[*U*(*π*_*r*_(*Q*_*λ*_*, *e*_*λ*_*))]/∂*Q*∂*e* > 0. After combining the above results, we obtain ∂*Q*_*λ*_*/∂*e*_*λ*_* > 0.

Proof Let *Q* = *Q*(*e*(*λ*), *λ*). Using the chain rule, we obtain

(A.21) $$\begin{array}{c} \frac{dQ_{\lambda }^{*}}{d\lambda }=\frac{\partial Q_{\lambda }^{*}}{\partial e_{\lambda }^{*}}\cdot \frac{de_{\lambda }^{*}}{d\lambda }+\frac{\partial Q_{\lambda }^{*}}{\partial \lambda }. \end{array}$$

By the implicit function theorem, from (31), we have

(A.22) 1φ(eλ∗)(p−v)  ×[(λ−1)(w−v)2f(q(Qλ∗,eλ∗)φ(eλ∗))+(p−v)2f(Qλ∗φ(eλ∗))]  ·∂Qλ∗∂λ+(w−v)F(q(Qλ∗,eλ∗)φ(eλ∗))=0.

From (A.22), ∂*Q*_*λ*_*/∂*λ* < 0. From Properties 1 and 2, *de*_*λ*_*/*dλ* < 0 and ∂*Q*_*λ*_*/∂*e*_*λ*_* > 0. After combining the above results, we then obtain *dQ*_*λ*_*/*dλ* < 0.

Proof of Lemma 7. {*e*_*T*_*, *Q*_*T*_*} satisfies the first-order conditions (6). By solving (6), we obtain the following:

(A.23) $$\begin{array}{c} \frac{g^{\prime }\left(e_{T}^{*}\right)}{\varphi^{\prime }\left(e_{T}^{*}\right)}=\left(p-v\right)\overset{F^{-1}(r)}{\underset{0}{\int }}xf\left(x\right)dx,\\ Q_{T}^{*}=\varphi \left(e_{T}^{*}\right)F^{-1}\left(r\right), \end{array}$$

where *r* = (*p* − *c*)/(*p* − *v*). Replacing *c* with *w* in (A.23), we obtain the optimal effort-quantity {*e*_0_*, *Q*_0_*} of the risk-neutral retailer. Based on our assumptions, it is easy to verify that *g*′(·)/*φ*′(·) is an increasing function. In addition, *F* ^−1^(·) is also an increasing function, which leads to the conclusion.
